# Digital vaccines for immunization equity: an approach to strengthen vaccine delivery and public trust in low- and middle-income countries

**DOI:** 10.1093/jamiaopen/ooag045

**Published:** 2026-04-09

**Authors:** Zhinya Kawa Othman, Mohamed Mustaf Ahmed, Olalekan John Okesanya, Shuaibu Saidu Musa, Don Eliseo Lucero-Prisno

**Affiliations:** Department of Pharmacy, Kurdistan Technical Institute, Sulaymaniyah, Kurdistan Region 46001, Iraq; Faculty of Medicine and Health Sciences, SIMAD University, Mogadishu 252, Somalia; Department of Public Health and Maritime Transport, Faculty of Medicine, University of Thessaly, Volos 38221, Greece; School of Global Health, Faculty of Medicine, Chulalongkorn University, Bangkok 10330, Thailand; Department of Global Health and Development, London School of Hygiene and Tropical Medicine, London WC1E 7HT, United Kingdom; Center for Research and Development, Cebu Normal University, Cebu 6000, Philippines; Center for University Research, University of Makati, Makati City 1218, Philippines

**Keywords:** vaccines, vaccine confidence, immunization equity, LMICs, SMS reminders, electronic immunization registries

## Abstract

**Background:**

Global immunization efforts still face major inequities and declining vaccine confidence, leaving millions of children in low- and middle-income countries unvaccinated or under-vaccinated.

**Objectives:**

This article aims to discuss “digital vaccines,” including SMS reminders, mobile apps, electronic immunization registries, gamification, and virtual reality education, as practical complements to routine immunization services.

**Results:**

Using an organizing framework focused on access, equity, and trust, we highlight how digital tools can reduce missed appointments, strengthen follow-up for zero-dose children, improve data quality for planning, and support transparent and culturally responsive communication to counter misinformation. We also outline the barriers that limit equitable impact, including digital divides, gender gaps in phone access, fragmented information systems, limited financing, and concerns about data governance. Many children in poorer countries still do not get the vaccines they need. Some families live too far from clinics. Others do not trust vaccines or the health system. This article looks at how digital tools can help more children get vaccinated. These tools include text message reminders, phone apps, online health records, digital games, and virtual reality lessons. Text reminders help parents remember vaccine dates. Online records help health workers find children who missed their vaccines. Digital games teach people why vaccines are safe. These tools can also help planners know how many vaccines are needed and where to send them. They can share clear, respectful health messages and fight false claims about vaccines. But not everyone can use these tools. Some people do not have smartphones or internet access. Women, who often care for children, may not have their own phones. There are also worries about keeping personal data safe and paying for these systems.

**Conclusions:**

We propose implementation principles that emphasize inclusive design, interoperability, privacy safeguards, and hybrid online and offline delivery models. We suggest that digital tools should be easy to use for all, keep private data safe, and work well with other health systems. Where there is no internet, non-digital options should also be offered. With the right support, these tools can help make sure all children get their vaccines.

## Introduction

Global immunization efforts continue to face challenges in achieving equitable coverage, with millions of children, primarily in low- and middle-income countries (LMICs), remaining unvaccinated or under-vaccinated. Although the Sustainable Development Goals (SDGs), adopted in 2015, aim to ensure universal health coverage (UHC) and equitable access to vaccines by 2030, significant disparities persist, particularly in these regions.^1^^,^^2^ In 2023, 14.5 million infants missed their first dose of the diphtheria-tetanus-pertussis (DTP) vaccine, and another 6.5 million were only partially vaccinated, highlighting major gaps in immunization and healthcare access worldwide. Nearly 60% of these 21 million under-vaccinated children are concentrated in 10 countries, including Afghanistan, Angola, the Democratic Republic of the Congo (DRC), Ethiopia, India, Indonesia, Nigeria, Pakistan, Sudan, and Yemen.^3^ Vaccination coverage is influenced by physical factors such as cost, supply, logistics, and access, as well as attitudinal factors such as public perception.^4^

Simultaneously, public confidence in vaccines has been threatened by misinformation and erosion of trust in vaccine safety and efficacy, the competence of healthcare professionals who offer vaccines or guidance, and the overall health system.^5^ For example, a meta-analysis found that only 49% of parents in LMICs were willing to vaccinate their children against COVID-19, primarily because of concerns about vaccine safety and efficacy.^6^ Public trust significantly influences vaccine uptake, as shown in a McKinsey & Company survey in which only 59% of respondents in the DRC were willing to receive a COVID-19 vaccine, mainly due to safety concerns and distrust in public messaging.^7^ Similar mistrust fueled hesitancy in Mayan communities in Guatemala, who rejected vaccine teams amid deep-rooted distrust of government health initiatives, reflecting historical neglect and perceived inequality.^8^ Recognizing the global nature of this issue, the World Health Organization (WHO) listed vaccine hesitancy as one of the top 10 global health threats, stressing that trust in vaccines, health professionals, and policymakers is foundational to successful immunization programs.

These factors contribute to unequal vaccine access and may sustain low vaccination rates. To address this issue, the Immunization Agenda 2030 (IA2030) aims to ensure that people of all ages benefit fully from vaccines, with a strong emphasis on reaching under-immunized and zero-dose children in marginalized and underserved communities.^9^ In parallel, digital vaccines, referring to digital immunization support tools such as mobile apps, SMS reminders, and electronic registries, are gaining attention for their potential to strengthen vaccine delivery systems and improve communication with communities.^10^ These tools can enhance timely immunization, reduce missed opportunities for vaccination, and support public trust through improved follow-up, information sharing and service coordination. This perspective explores how digital vaccines and broader digital health tools can be used to improve vaccine uptake and promote equity in low-resource settings, highlighting key implementation challenges and practical recommendations.

## Impact of digital vaccines on delivery and public trust

**Figure 1:**
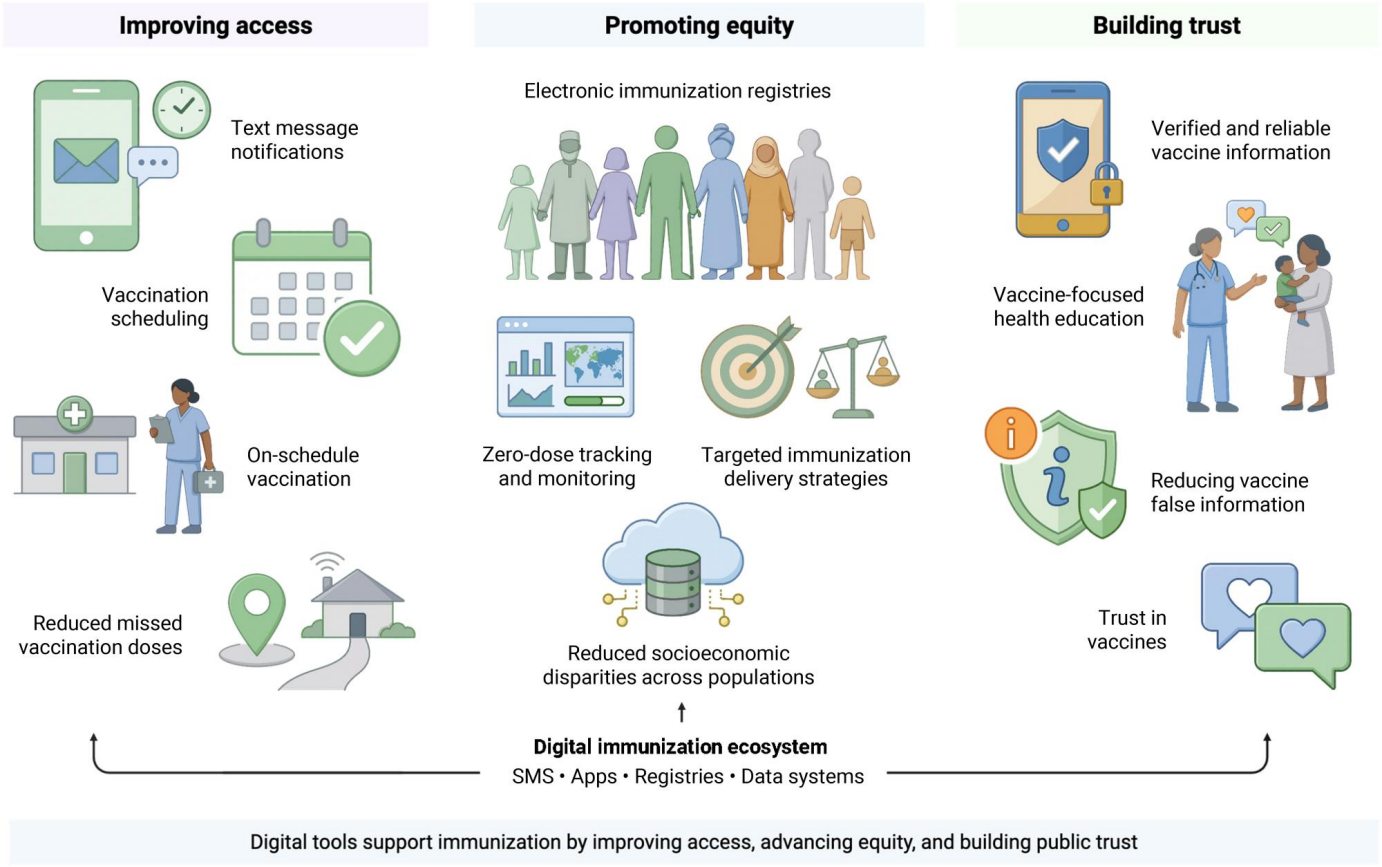
Organizing framework linking digital tools to immunization access, equity, and trust. Created in BioRender. https://BioRender.com/du54ldw.

It is essential to explore how digital tools support immunization efforts through an organizing framework built on 3 key pillars: improving access, promoting equity, and building trust (Figure 1). This framework is presented to structure the discussion in this perspective and draws on IA2030 and WHO guidance on vaccine uptake and hesitancy (including the “3Cs” and the Behavioral and Social Drivers of Vaccination approach).^9^^,^^11^^,^^12^ Digital interventions can play a major role in expanding access by streamlining vaccine delivery, such as sending reminders to caregivers, easing logistics, and providing real-time data for decision-making, which leads to improved health literacy.^13^

For example, MomsTalkShots is a personalized educational app developed to enhance maternal and infant vaccination uptake. The app was well received across diverse demographic groups, including vaccine-hesitant individuals, significantly improved perceived vaccine knowledge, with 62% of users reporting high knowledge compared with 50% in the control group; among participants initially uncertain about vaccination, high perceived knowledge increased to 47% versus 12% in controls.^14^^,^^15^ Furthermore, digital vaccines can promote equity by better targeting underserved populations and reducing gaps in service delivery.

For example, electronic immunization registries (EIRs), which are secure, population-based digital systems that record individualized vaccine dose data across multiple providers to support timely and accurate vaccine delivery, allow for aggregated analysis to guide immunization program planning and monitoring and follow up with children who missed vaccines, thus narrowing coverage disparities.^16^ EIRs are further tailored to address specific health system challenges affecting vaccination coverage and equity, such as inaccurate data, poor tracking, and weak coordination. Their effectiveness depends on design features, with some including stock management and others relying on interoperability with other digital tools to overcome broader system barriers.^17^

Although vaccination is an essential component of primary prevention efforts in public health, vaccine hesitancy remains a global concern driven by various factors, such as religious beliefs, past negative experiences with foreign healthcare systems, adverse effects of vaccines on LMICs, and safety concerns in high-income nations. Thus, digital solutions can help rebuild trust in vaccines and health systems by improving communication, combating misinformation, and increasing transparency in the health system. They also create a supportive environment that informs and reassures individuals, leading to improved vaccine uptake and timely immunization.^18^

## Digital vaccine applications in LMICs

In the wake of the modern digital era, online content can shape people’s motivation and decision-making by affecting how they evaluate vaccine risks and benefits. Information sources vary greatly, ranging from accurate government websites to unverified blogs and social-media posts. While social media enables open communication, it has also become a major source of vaccine misinformation and anti-vaccine sentiment, fueling the skepticism and confusion that contribute to hesitancy.^19^ Studies have shown that interventions based on behavior change theories and evidence-based principles are more effective than those without a theoretical basis. Thus, theory-based approaches offer valuable insights into vaccine hesitancy.^20^ These theories include the Health Belief Model, which focuses on how people’s beliefs influence their health behaviors; the Theory of Planned Behavior, which considers attitudes, social pressure, and perceived control over actions; and Social Cognitive Theory, which highlights learning through observation and building confidence in one’s ability to act. These theories can help guide the design and delivery of vaccine communication and engagement strategies to strengthen knowledge and confidence across populations.^20^  Table 1 provides illustrative examples of digital tools used in LMICs and their potential contributions to immunization equity.

**Table 1:** Examples of digital vaccine tools in LMICs and their role in enhancing immunization equity.

**Digital tools** ^a^	Application	Country(s)	Equity impact	Reference(s)
**Digital gamification**	Community co-developed digital games to counter misinformation and build resistance to anti-vaccine narratives	Ghana	Built trust by involving local users in design and improved digital health equity through participatory methods	^22^
**Electronic immunization registries (EIRs)**	Tracks individual immunization records, improves data accuracy, and facilitates targeted outreach to under-immunized populations	Tanzania	Strengthen immunization services in underserved areas and improved follow-up for zero-dose and missed children	^16^
**Virtual reality (VR)**	Provides culturally and linguistically tailored vaccine education to improve acceptance among refugee populations	Somalia	Enhanced understanding and trust in vaccines among marginalized, linguistically diverse communities to reduces hesitancy	^24^
**Short message service (SMS)**	Delivered personalized SMS reminders to parents for infant immunization appointments	Guatemala	Improved timely infant vaccine uptake through reaching caregivers in low-resource and rural areas	^25^

a The examples in this table were selected as illustrative case examples based on prominence in the literature, diversity of settings, and representation of different digital approaches. They are not intended to represent a comprehensive review of all digital immunization interventions in LMICs.

A study showed that gamification (incorporating game-like features such as rewards, challenges, interactive feedback, and engaging interfaces into contexts outside of traditional gaming) can increase predictors of vaccine uptake, such as knowledge, attitudes, beliefs, behaviors, and vaccination intention, which leads to enhanced vaccination coverage.^21^ A pilot study in Ghana tested the effectiveness of a digital game called Cranky Uncle Vaccine, which was designed to counter vaccine misinformation through psychological inoculation. Notably, 53% of those initially hesitant became more open to vaccination after playing, which showed the game’s effectiveness in promoting vaccine confidence and countering false claims.^22^

In addition, EIRs contribute to improving immunization programs through better data and resource planning to enhance vaccination coverage. Hence, Tanzania introduced this system in 15 of its regions. Consequently, EIRs offer significant benefits beyond tracking vaccine coverage by enabling detailed analyses of service delivery, identifying under-immunization risks, and pinpointing facility-level issues.^16^ Utilizing further innovative approaches such as immersive virtual reality (VR) for vaccine advocacy may help to reach younger people, who are more likely to be hesitant about vaccination due to a lower likelihood of suffering from a severe course of the disease. Research has shown that immersive, gamified VR experiences significantly boost vaccination intentions by allowing users to personally experience the collective benefits of immunization. In a randomized trial, VR exposure increased vaccination intention by 9.3 points among participants with less-than-maximal baseline intention, compared with a 3.3-point increase using text-and-image messaging alone, demonstrating a substantially stronger effect of immersive delivery.^23^

These findings suggest that the unique impact of VR may stem from its emotional engagement and sense of presence. Another study developed a culturally and linguistically tailored VR vaccination education platform for Somali refugees using community-based participatory research. The outcome highlighted that flexible, community-engaged VR tools can improve vaccination education and may be adaptable for other culturally diverse populations.^24^ Likewise, as global mobile phone access continues to increase, short message service (SMS) texts have shown significant potential for improving health, including in LMICs, especially for treatment adherence. Studies have shown that childhood immunization reminder systems can be effectively adapted to SMS platforms, improving vaccination rates in both high-income countries, such as the United States, and LMICs, such as Guatemala, Bangladesh, India, and Kenya.^25^

## Challenges and recommendations

Despite the great potential of digital tools to improve immunization in LMICs, significant challenges hinder their equitable and sustainable implementation. Digital divides are a major concern, as populations with low vaccination rates often lack access to the tools needed to support them, such as smartphones, Internet connectivity, or digital literacy, especially among rural, poor, and marginalized communities. Gender disparities further limit access, with women, often primary caregivers, being less likely to own or control mobile devices. Without inclusive design, digital interventions risk reinforcing existing inequalities.^26^ To ensure a broad reach, digital strategies should be complemented by offline approaches and efforts to overcome structural barriers. For a lasting impact, countries can invest in governance frameworks that support integrated and reusable digital platforms instead of isolated apps. Funding gaps further hinder the integration of digital systems into immunization programs, as resources are often prioritized for vaccine procurement and delivery staff, with digital tools viewed as nonessential.^13^

To secure domestic and international funding, it is crucial to demonstrate the value of digital health through evidence such as cost-effectiveness studies, which may be limited. Innovative financing models, such as public-private partnerships or performance-based grants that tie funding to digital system use or measurable outcomes, can support broader adoption. Additionally, in many LMICs, health information systems have been developed independently for different areas, such as immunization programs, logistics, and maternal health, resulting in isolated systems. Integrating these systems is complex and requires shared data standards, a collaborative approach to integration, and, at times, difficult negotiations with software providers or workarounds for legacy systems.^27^ Lastly, to promote equity and trust in digital vaccine programs, key principles should guide implementation, such as inclusive design that meets the needs of marginalized groups through accessible communication and user-centered approaches; interoperability and integration to ensure systems are connected, efficient, and avoid duplication; and strong data governance and privacy protections to build public trust through clear regulations, secure systems, and community involvement in data oversight.

## Conclusion

Digital vaccines serve as a potent avenue for enhancing vaccine equity and trust, particularly in LMICs. When seamlessly integrated into healthcare systems and designed to meet people’s needs, they can improve timely access to vaccines, data accuracy and public trust. From SMS notifications to comprehensive national digital registries, digital solutions bolster immunization at every level but rely on a robust infrastructure, effective policies, and active community involvement. As nations embrace digital immunization strategies, a proactive research agenda is essential to close the knowledge gaps and maximize the benefits. Key areas of focus include evaluating the long-term impacts of digital tools on coverage and equity and whether their advantages are sustained or need reinforcement. Research should also examine whether these tools help reduce equity disparities and enhance vaccine confidence by employing methods such as surveys, trust indices, and comparative studies. Although technology itself is not a cure-all, human initiatives to address long-standing challenges are imperative. Through ongoing investment and inclusive application, digital vaccines can significantly contribute to achieving universal immunization and advancing global health objectives.

## Date availability

Data sharing does not apply to this article, as no datasets were generated or analyzed.
